# Human odontoblast-like cells produce nitric oxide with antibacterial activity upon TLR2 activation

**DOI:** 10.3389/fphys.2015.00185

**Published:** 2015-06-23

**Authors:** Jean-Christophe Farges, Aurélie Bellanger, Maxime Ducret, Elisabeth Aubert-Foucher, Béatrice Richard, Brigitte Alliot-Licht, Françoise Bleicher, Florence Carrouel

**Affiliations:** ^1^Institut de Génomique Fonctionnelle de Lyon, UMR5242 Centre National de la Recherche Scientifique/ENS/Université Lyon 1, Equipe Physiopathologie des OdontoblastesLyon, France; ^2^Faculté d'Odontologie, Université Lyon 1, Université de LyonLyon, France; ^3^Hospices Civils de Lyon, Service de Consultations et Traitements DentairesLyon, France; ^4^Laboratoire de Biologie Tissulaire et Ingénierie Thérapeutique, Institut de Biologie et Chimie des Protéines, UMR5305 Centre National de la Recherche Scientifique/Université Lyon 1Lyon, France; ^5^Faculté d'Odontologie, Centre de Recherche en Transplantation et Immunologie, INSERM UMR1064, Université de NantesNantes, France

**Keywords:** tooth, odontoblast, caries lesion, dental pulp regeneration, nitric oxide, toll-like receptor 2, *Streptococcus mutans*

## Abstract

The penetration of cariogenic oral bacteria into enamel and dentin during the caries process triggers an immune/inflammatory response in the underlying pulp tissue, the reduction of which is considered a prerequisite to dentinogenesis-based pulp regeneration. If the role of odontoblasts in dentin formation is well known, their involvement in the antibacterial response of the dental pulp to cariogenic microorganisms has yet to be elucidated. Our aim here was to determine if odontoblasts produce nitric oxide (NO) with antibacterial activity upon activation of Toll-like receptor-2 (TLR2), a cell membrane receptor involved in the recognition of cariogenic Gram-positive bacteria. Human odontoblast-like cells differentiated from dental pulp explants were stimulated with the TLR2 synthetic agonist Pam2CSK4. We found that *NOS1*, *NOS2*, and *NOS3* gene expression was increased in Pam2CSK4-stimulated odontoblast-like cells compared to unstimulated ones. *NOS2* was the most up-regulated gene. NOS1 and NOS3 proteins were not detected in Pam2CSK4-stimulated or control cultures. NOS2 protein synthesis, NOS activity and NO extracellular release were all augmented in stimulated samples. Pam2CSK4-stimulated cell supernatants reduced *Streptococcus mutans* growth, an effect counteracted by the NOS inhibitor L-NAME. *In vivo*, the *NOS2* gene was up-regulated in the inflamed pulp of carious teeth compared with healthy ones. NOS2 protein was immunolocalized in odontoblasts situated beneath the caries lesion but not in pulp cells from healthy teeth. These results suggest that odontoblasts may participate to the antimicrobial pulp response to dentin-invading Gram-positive bacteria through NOS2-mediated NO production. They might in this manner pave the way for accurate dental pulp healing and regeneration.

## Introduction

Odontoblasts are neural crest-derived, highly specialized mesenchymal cells organized as a densely packed layer at the periphery of the loose connective tissue situated in the center of the tooth, the dental pulp. Their main functions are the synthesis, extracellular deposition and mineralization of a collagen-rich matrix to form the dentin tissue that surrounds the dental pulp and underlies the surface enamel. Recent data have indicated that odontoblasts may also have functions not related to dentinogenesis (Bleicher et al., 2015). Indeed, because of their specific location at the pulp-dentin interface and the entrapment of their long cell process in dentin, they become exposed to dentin-invading oral bacteria during the carious process (Love and Jenkinson, 2002). Odontoblasts are the first cells encountered by tooth-invading pathogens and they have been suggested to initiate pulp immune and inflammatory responses to these pathogens (Durand et al., 2006; Veerayutthwilai et al., 2007). These responses may eliminate the insult and block the route of infection. Unchecked, bacterial invasion results in irreversible pulp inflammation then necrosis, and dissemination of potentially lethal microorganisms may occur throughout the body (Farges et al., 2009). In parallel, dentin formation appears greatly disturbed (Bjorndal and Mjör, 2001; Durand et al., 2006). Several lines of evidence now support the notion that it is only when pulp infection and inflammation are under control that dentinogenesis-based pulp regeneration will occur. In this context, further studies are needed to elucidate the odontoblast response to cariogenic bacteria in order to design new antibacterial therapeutics that will reduce dental pulp inflammation while promoting tissue healing and regeneration (Farges et al., 2013; Cooper et al., 2014).

Studies that aimed at elucidating the triggering of dental pulp immunity by odontoblasts have mostly focused on gram-positive bacteria, because these largely dominate the microflora in initial and moderate dentin caries lesions (Love and Jenkinson, 2002; Hahn and Liewehr, 2007). In particular, odontoblast-like cells were found to be responsive to lipoteichoic acid (LTA), a Gram-positive bacteria component recognized at cell surface through the pattern recognition receptor (PRR) Toll-like receptor-2 (TLR2). Engagement of odontoblast TLR2 by LTA up-regulates TLR2 itself and nucleotide-binding oligomerization domain 2, a cytosolic PRR. It also stimulates the production of the proinflammatory chemokines and cytokines CCL2, CXCL1, CXCL2, CXCL8, CXCL10, and interleukin (IL)-6, and the recruitment of immature dendritic cells (Durand et al., 2006; Staquet et al., 2008, 2011; Farges et al., 2009, 2011; Keller et al., 2010, 2011). IL-10, a cytokine that plays a central role in limiting host immune responses to pathogens, was also up-regulated. Similar, effects were observed when using Pam2CSK4, a synthetic diacylated lipopeptide analog that specifically binds TLR2. Of note, the response obtained with Pam2CSK4 was always higher than with LTA. However, no additional cytokine was induced by Pam2CSK4 compared to LTA, suggesting that odontoblasts secrete a limited set of proinflammatory factors when challenged with Gram-positive bacteria. We also observed that the lipopolysaccharide (LPS)-binding protein (LBP) was up-regulated by Pam2CSK4 (our unpublished results) and that it decreased TLR2 activation and proinflammatory cytokine production in odontoblast-like cells (Carrouel et al., 2013).

If the role of odontoblasts in the triggering and control of dental pulp immunity begins to be elucidated, their involvement in the direct fight against dentin-invading bacteria is far less known. Among antibacterial agents putatively produced by odontoblasts, nitric oxide (NO) has recently received particular attention. NO is a highly diffusible, gaseous free radical generated by nitric oxide synthases (NOS) through the conversion of L-arginine to L-citrulline. Three NOS isoforms have been identified so far: two are constitutively expressed at low levels, NOS1 (neuronal NOS) and NOS3 (endothelial NOS), whereas one is produced upon cell stimulation by microorganisms or proinflammatory cytokines, NOS2 (inducible NOS). NOS1 and NOS3 participate to normal tissue functions by constitutively synthesizing very small amounts (picomolar to nanomolar levels) of short acting NO (seconds to minutes). NOS2 is the enzyme responsible for high NO production in infection settings (Arthur and Ley, 2013). It is predominantly regulated at the transcriptional level and is involved in antibacterial defense by producing large amounts of NO, up to micromolar levels, for sustained periods of time (hours to days) (Nathan, 1992; Nussler and Billiar, 1993; MacMicking et al., 1997; Bogdan, 2001; Coleman, 2001; Guzik et al., 2003). Previous studies indicated that the *NOS2* gene was not or weakly expressed in healthy dental pulps, but was sharply up-regulated in inflamed ones (Law et al., 1999; Di Nardo Di Maio et al., 2004; Kawashima et al., 2005; Korkmaz et al., 2011). Human odontoblasts showed a marked immunoreactivity for 3-nitrotyrosine (a biomarker for NO-derived peroxinitrite) in inflamed pulp, suggesting that these cells release NO upon NOS2 activation. NO production by odontoblasts might be an important defense mechanism against dentin-invading oral microorganisms because it inhibits *Streptococcus mutans* growth (Silva-Mendez et al., 1999). Despite these important findings, no direct evidence was brought so far that odontoblasts produce NO amounts bactericidal for caries-related, gram-positive microorganisms. Therefore, the aim of our study was to investigate, in a culture model of human odontoblasts differentiated *in vitro*, if these cells are able to produce NO with antibacterial activity upon TLR2 engagement. We first studied the effects of Pam2CSK4 stimulation on odontoblast *NOS1, NOS2*, and *NOS3* gene expression. We also examined NOS1, NOS2, and NOS3 protein synthesis, NOS intracellular activity and NO secretion upon Pam2CSK4 stimulation. We then assessed the antibacterial effect of odontoblast-derived NO by analyzing the growth of *Streptococcus mutans* bacteria in the presence of TLR2-activated or control odontoblast-like cell culture supernatants. Finally, expression of NOS2 transcript and protein was investigated *in vivo* in healthy and bacteria-challenged inflamed dental pulps.

## Materials and methods

### Reagents

The synthetic diacylated lipopeptide analog Pam2CSK4 was from InvivoGen (San Diego, CA, USA). The mouse anti-NOS2 monoclonal antibody (clone 2D2-B2) was from R&D Systems Europe (Lille, France). Rabbit anti-NOS1 monoclonal (EP1855Y) and anti-NOS3 polyclonal antibodies were from Abcam (Cambridge, UK). The mouse anti-GAPDH monoclonal antibody (clone 8C2) was from Santa Cruz Biotechnology (Santa Cruz, CA USA). The mouse immunoglobulin G1 isotype control antibody (clone MOPC-21) and the arginine analog NOS inhibitor NG-nitro-L-arginine methyl ester (L-NAME) were from Sigma-Aldrich (St Louis, MO, USA).

### Dental pulp samples

Healthy and decayed human teeth were collected with informed consent of the patients or their parents, in accordance with the World Medical Association's Declaration of Helsinki and following a protocol approved by the local ethics committee. Healthy pulps were taken from impacted third molars. Inflamed pulps were taken from decayed erupted molars with clinical features of acute pulpitis (deep dentin caries lesions, severe spontaneous dental pain for 12–24 h, no sensitivity to vertical or horizontal percussion, lack of periapical lesions) and in the absence of anti-inflammatory treatment.

### Cell culture and treatments

Odontoblast-like cells were differentiated from dental pulp explants obtained from clinically healthy, impacted human third molars as previously described (Couble et al., 2000), then used for stimulation experiments. For detection of *NOS* gene expression, NOS protein production, NOS activity and NO extracellular levels, cells were cultured for the indicated times in the absence (controls) or in the presence of 10 μg/mL Pam2CSK4. To determine the antibacterial effect of odontoblast-like cell-derived NO on *Streptococcus mutans* bacteria, cells were treated with 10 μg/mL Pam2CSK4 for 24 h, and then culture media were collected and frozen until further use (see below). Some cultures were pretreated for 1 h with 0.5 or 1 mmol/L L-NAME prior to addition of Pam2CSK4 to confirm NO involvement in the effect observed.

### Reverse transcription-polymerase chain reaction

RNA extraction and reverse transcription were performed from Pam2CSK4-stimulated and control odontoblast-like cells and from healthy and inflamed dental pulp samples as described (Keller et al., 2010; Farges et al., 2011). Real-time polymerase chain reaction (PCR) was performed in a CFX96 Real-Time PCR Detection System with the Fast Start Master SYBR Green I kit (Bio-Rad Laboratories, Hercules, CA, USA) according to the manufacturer's specifications. The cyclophilin A housekeeping gene (*PPIA*) was used for sample normalization. Gene-specific primer sequences for *NOS1*, *NOS2*, *NOS3*, and *PPIA* are listed in Table 1. Annealing temperature was 65°C for all primer pairs. All runs were performed in duplicate. For each target gene, relative expression was determined after normalization using the Bio-Rad CFX Manager software. Results were expressed as fold change values relative to control odontoblast-like cell cultures for *in vitro* analyses and to healthy pulp samples for *in vivo* analyses.

**Table 1:** **Primers used for PCR analysis**.

**Gene**	**Forward primer**	**Reverse primer**	**Amplicon size (bp)**
*NOS1*	CTGATACCAAAAGCCTCTCT	ATCTGAGCCTAACAATCTGG	76
*NOS2*	ACAGGCTCGTGCAGGACTCA	CACGGCTGGATGTCGGACTT	126
*NOS3*	CATGAGCACTGAGATCGGCA	CCAGGATGTTGTAGCGGTGA	59
*PPIA*	GGATTGCTTGAGCCTAGAGTGA	CCTCTGCCTACCTTTGAGACAC	87

### Protein extraction and western blotting

Cells were washed twice with PBS and overlaid with ice-cold RIPA buffer (Sigma-Aldrich) supplemented with a protease inhibitor cocktail (P8340, dilution 1:100; Sigma-Aldrich). After 5 min on ice, cells were scraped and insoluble material was removed by centrifugation at 12,000 g for 10 min at 4°C. Total proteins were quantified by a Bradford assay (Coomassie Protein Assay Reagent; Pierce, Thermo Fischer Scientific Inc., Rockford, IL, USA). Proteins were concentrated by precipitation for NOS isoform detection. After addition of 4 volumes of cold acetone and incubation for at least 1 h at −20°C, pellets were collected by centrifugation at 12,000 g for 10 min at 4°C, air-dried and dissolved in reducing Laemmli sample buffer. Identical amounts of proteins were loaded for Pam2CSK4-stimulated and control samples. Positive controls for NOS1 (neuronal NOS) and NOS3 (endothelial NOS) were extracts from mouse whole brain and human umbilical vein endothelial cells (HUVEC), respectively. HUVEC were kindly provided by Dr Laurent Müller (CIRB CNRS UMR7241 - INSERM U1050, Collège de France, Paris). Proteins were resolved by 8.5% sodium dodecyl sulfate polyacrylamide gel electrophoresis and transferred to PVDF membranes (Millipore, Molsheim, France). Membranes were probed with anti-NOS1 (diluted 1:1000), anti-NOS2 (1:400), anti-NOS3 (1:1000) or anti-GAPDH (1:2000) and incubated with HRP- or alkaline phosphatase-conjugated anti-rabbit or anti-mouse IgGs (1:1000; Cell Signaling). Bound antibodies were detected on x-ray films by using Immunstar PA, Immunstar HRP or WesternC chemiluminescent substrates (Bio-Rad Laboratories).

### Detection of NOS activity and extracellular NO production

NOS activity and NO production by Pam2CSK4-stimulated and control odontoblast-like cells were quantified in cell lysates and culture media by using an Ultrasensitive Colorimetric NOS Assay kit and a Nitric Oxide Colorimetric Assay kit (Oxford Biomedical Research, Oxford, MI, USA), respectively, according to the manufacturer's specifications. Cells were lysed in Beadlyte® Cell Signaling Universal Lysis Buffer (Millipore). NO is highly labile, with a half-life in the order of seconds, and these kits are based on the colorimetric quantitation of nitrite (NO^−^_2_), the stable end product of NO oxidation, using Griess reagent. They include the conversion of nitrate to nitrite by NADH-dependent enzyme nitrate reductase before nitrite measurement, in order to provide for accurate determination of total NO production. Absorbance values were read at 540 nm using a microplate reader. NO/nitrite sample concentrations were determined from a sodium nitrite standard curve. Intracellular NO/nitrite concentration was expressed as μmoles/μg of proteins (determined using a Bradford protein assay).

### *Streptococcus mutans* growth assessment

The *Streptococcus mutans* strain (CIP 103220) was purchased from Institut Pasteur (Paris, France). Bacteria were cultured in Brain Heart Infusion broth (Biomérieux, Marcy-L'Etoile, France) at 37°C for 24 h. After centrifugation at 2500 rpm for 5 min, the supernatant was discarded and the bacterial pellet resuspended in water for determination of the bacteria number with McFarland Standard (Biomérieux). Samples containing 10^8^ bacteria were then taken and centrifuged at 15,000 rpm for 5 min. Bacterial pellets were resuspended in 100 μL odontoblast-like cell culture supernatants, then maintained at 37°C for 15, 30, 60, or 90 min, the later time being just before the end of the bacteria growth phase as determined by our preliminary experiments (not shown). Bacteria-containing samples were then plated onto Columbia agar supplemented with 5% defibrinated horse blood. Bacterial cultures were performed in anaerobic conditions (GENbox anaer, Biomérieux) for 5 days at 37°C, then *S. mutans* colonies were counted.

### Immunohistochemistry

Healthy teeth and carious ones with inflamed pulps were fixed in 4% paraformaldehyde-phosphate-buffered saline solution for 7 days, demineralized in 10% acetic acid for 4 months, and routinely treated for paraffin embedding. Eight-micrometer serial sections were then cut, deparaffinized, and rehydrated. Endogenous peroxidase was blocked by incubation in 0.3% hydrogen peroxide for 15 min at room temperature. Sections were incubated for antigen retrieval in 10 mmol/L citrate buffer (pH 6.0, 98°C) for 10 min and then blocked with normal horse serum for 45 min at room temperature. Sections were then incubated with 12.5 μg/mL anti-NOS2 antibody overnight at 4°C. Staining controls were performed by using a mouse immunoglobulin G1 isotype. Antibody detection was performed by using a Vectastain Elite ABC kit (Vector Laboratories, Burlingame, CA, USA) according to the manufacturer's protocol, peroxidase being localized with diaminobenzidine.

### Statistical analysis

Results were expressed as mean values ± standard deviation obtained from different odontoblast-like cell cultures or tooth pulps originating from human third molars obtained from different donors. Statistical analysis was performed with a paired *t*-test. A *p* < 0.05 was considered significant.

## Results

### Pam2CSK4 increases *NOS1, NOS2*, and *NOS3* gene expression and NOS2 protein synthesis in odontoblast-like cells

**Figure 1:**
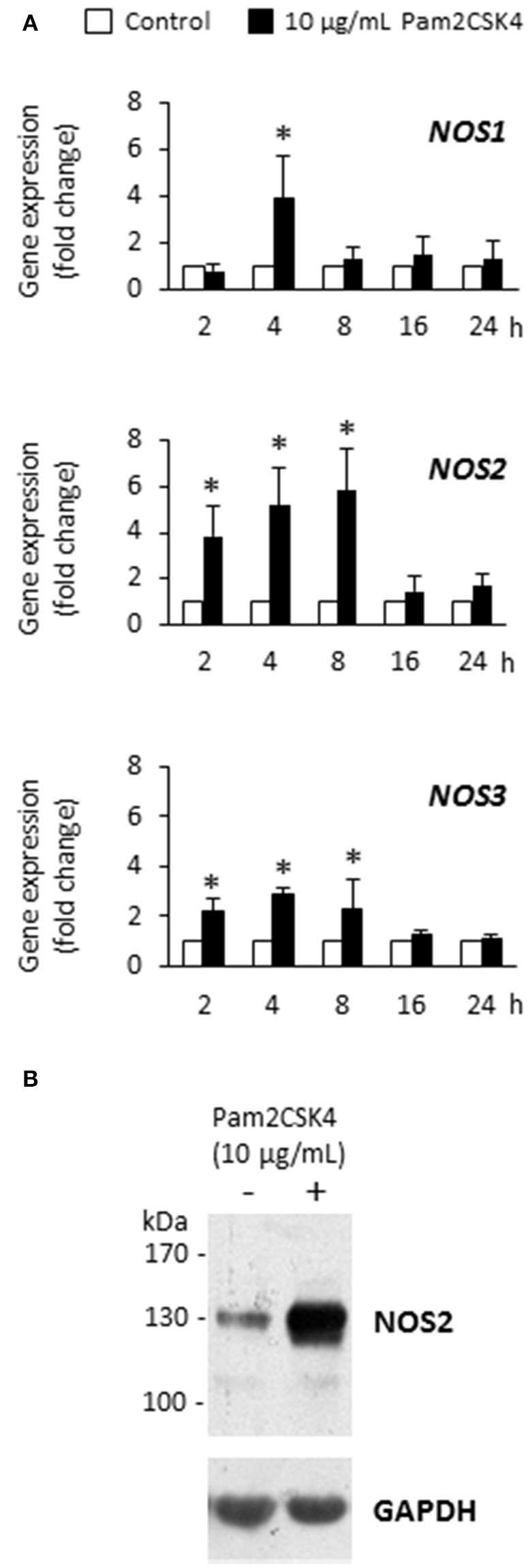
**Pam2CSK4 increases *NOS1*, *NOS2*, and *NOS3* gene expression and NOS2 protein synthesis in odontoblast-like cells. (A)** Analysis of *NOS1*, *NOS2*, and *NOS3* gene expression after cell challenge with 10 μg/mL Pam2CSK4 for the indicated times. The three genes were significantly up-regulated upon Pam2CSK4 stimulation, *NOS2* being the most up-regulated one (*n* = 4). ^*^p < 0.05. **(B)** At the protein level, NOS2 synthesis was clearly increased after 8 h of odontoblast-like cell stimulation with 10 μg/mL Pam2CSK4. The gel shown is representative of three independent experiments.

We previously found that the synthetic lipoprotein analog Pam2CSK4 at a 10 μg/mL concentration was particularly efficient to activate TLR2 in odontoblast-like cells and induced the production of proinflammatory cytokines and chemokines (Keller et al., 2010, 2011; Farges et al., 2011; Staquet et al., 2011). Therefore we used, in the present study, the same concentration to assess Pam2CSK4 effect on the expression of *NOS1*, *NOS2*, and *NOS3* genes by these cells. We observed that Pam2CSK4 significantly up-regulated the three genes tested (Figure 1A). The gene coding for the inducible NOS, *NOS2*, was the most up-regulated one, the increase being maximal after 8 h of cell stimulation. At the protein level, NOS2 was strongly increased after 8 h of Pam2CSK4 stimulation compared to control unstimulated cells (Figure 1B). NOS1 and NOS3 were not detected in control or Pam2CSK4-stimulated samples stimulated for 8 or 16 h (not shown).

### Pam2CSK4 increases NO production by odontoblast-like cells

**Figure 2:**
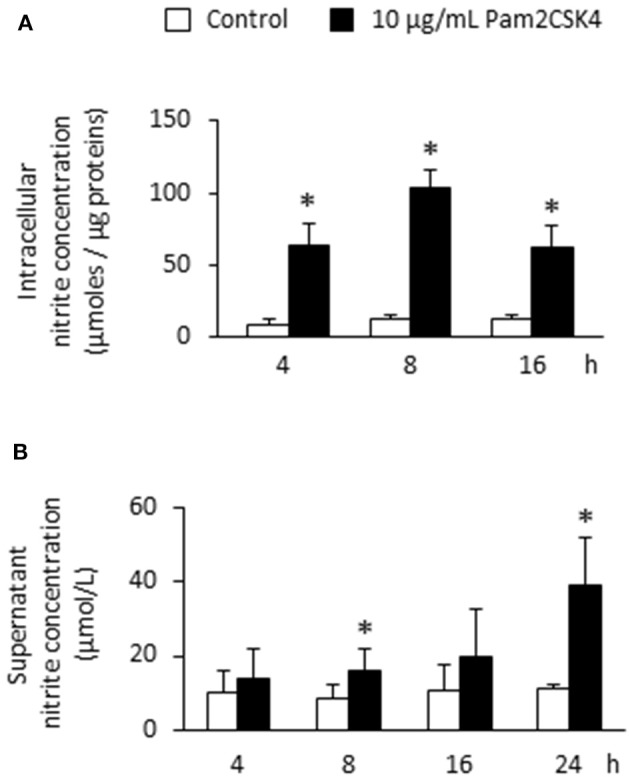
**Pam2CSK4 increases NOS activity and extracellular NO production by odontoblast-like cells. (A)** Analysis of intracellular NO by the measurement of nitrite concentration in cells stimulated with 10 μg/mL Pam2CSK4 for the indicated times. Production of intracellular nitrite was strongly increased in PAM2CSK4-stimulated samples, being maximal after 8 h (*n* = 5). **(B)** Determination of NO concentration in culture supernatants of odontoblast-like cells stimulated with 10 μg/mL Pam2CSK4. Nitrite progressively accumulated in the culture medium, reaching a concentration of approximately 40 μmol/L after 24 h of stimulation (*n* = 5). ^*^p < 0.05.

To determine whether NOS2 up-regulation upon Pam2CSK4 stimulation was accompanied by an increase in NOS activity and NO production, intracellular and extracellular NO concentrations were assessed by the measure of the NO degradation end-product nitrite. We observed a strong intracellular nitrite increase that was maximal after 8 h (Figure 2A). To assess NO diffusion to the extracellular compartment, we measured nitrite concentration in supernatants of Pam2CSK4-stimulated odontoblast-like cells. We found a progressive accumulation of nitrite in the culture medium reaching a concentration of approximately 40 μmol/L after 24 h, the longest stimulation time tested (Figure 2B). In unstimulated samples, the nitrite concentration remained low and constant with time, of approximately 10 μmol/L.

### Odontoblast-like cell-derived NO reduces *Streptococcus mutans* growth

**Figure 3:**
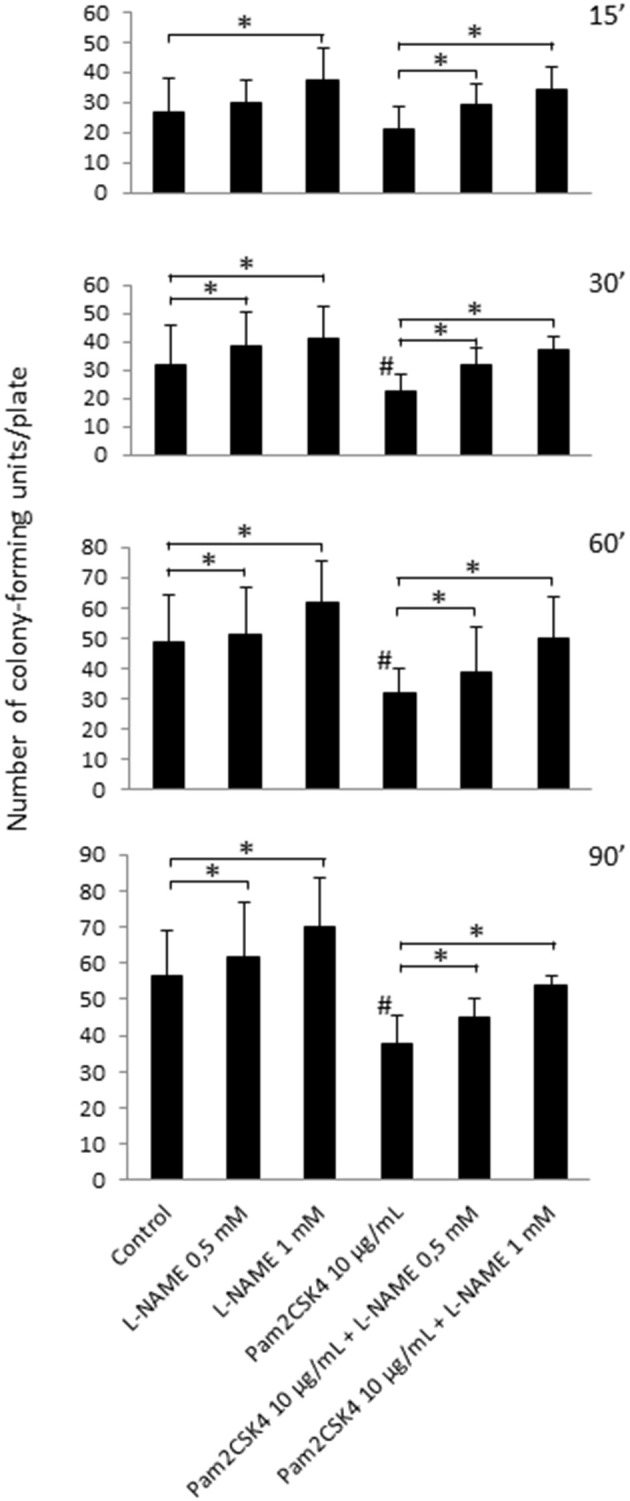
**NO released from unstimulated or Pam2CSK4-stimulated odontoblast-like cells alters *Streptococcus mutans* viability**. Analysis of *Streptococcus mutans* growth after contact of bacteria for 15, 30, 60, or 90 min with culture supernatants of cells challenged with 10 μg/mL Pam2CSK4 for 24 h in the presence or in the absence of L-NAME. The number of *Streptococcus mutans* colony-forming units was reduced when odontoblast-like cells were stimulated with Pam2CSK4 compared to unstimulated ones. Pretreatment with the NOS inhibitor L-NAME increased the number of *Streptococcus mutans* colony-forming units in both unstimulated and Pam2CSK4-stimulated samples (*n* = 6). ^#^*p* < 0.05 vs. control unstimulated cells. ^*^p < 0.05 vs. cultures without L-NAME.

Next, to evaluate the NO effect on the growth of cariogenic microorganisms, odontoblast-like cells were stimulated or not with 10 μg/mL Pam2CSK4 for 24 h with or without pretreatment with the NOS inhibitor L-NAME. Culture supernatants were collected and placed into contact with *Streptococcus mutans* bacteria for 15, 30, 60, or 90 min. In unstimulated samples, pretreatment with L-NAME increased the number of *Streptococcus mutans* colony-forming units, suggesting that NO from unstimulated odontoblast-like cells limits *Streptococcus mutans* growth (Figure 3). We observed that the number of *Streptococcus mutans* colony-forming units was clearly reduced in odontoblast-like cells stimulated with Pam2CSK4 compared to unstimulated ones, indicating a stronger antibacterial effect of culture supernatants from TLR2-activated cells. The decrease in NO production owing to increasing concentrations of L-NAME in Pam2CSK4-stimulated samples led to an augmentation of the number of *Streptococcus mutans* colony-forming units, thus indicating that NO production by odontoblast-like cells was indeed responsible for the observed slowdown of bacterial growth.

### NOS2 transcript and protein are up-regulated in inflamed pulps from decayed teeth

**Figure 4:**
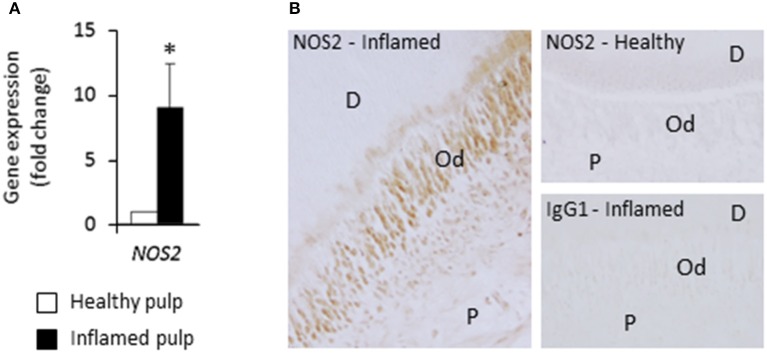
**NOS2 transcript and protein are up-regulated in inflamed pulps from decayed teeth compared to healthy ones. (A)** Analysis of *NOS2* gene expression in healthy and inflamed pulps with real-time RT-PCR (*n* = 3). ^*^p < 0.05. **(B)** Immunohistochemical localization of NOS2 protein in healthy and bacteria-challenged inflamed pulps. No NOS2 staining was found in healthy sample, but odontoblasts and subodontoblast cells were stained in inflamed pulp beneath the caries lesion. D, dentin; Od, odontoblast layer; P, pulp. Data shown are representative of results obtained from independent experiments performed with two healthy and two carious teeth.

To assess the *in vivo* relevance of these findings and determine whether NOS2 is expressed in odontoblasts, NOS2 transcript and protein were examined in healthy dental pulps and inflamed samples from carious teeth. We found that the *NOS2* gene was strongly up-regulated in inflamed pulps compared to healthy ones (Figure 4A). NOS2 protein was clearly detected by immunostaining in odontoblasts and subodontoblast cells in the inflamed area, but not in cells in the non-inflamed area far from the lesion (not shown) or in healthy pulps (Figure 4B). Staining controls performed by using the mouse immunoglobulin G1 isotype were negative.

## Discussion

Odontoblasts situated at the dental pulp periphery could play a role in the initiation of the tissue defense against dentin-invading Gram-positive oral bacteria through their ability to recognize pathogens and to produce proinflammatory cytokines and chemokines (Farges et al., 2013). In this context, chemokine secretion from odontoblasts may induce accumulation of immune cells at the pulp-dentin interface, including antigen-presenting immature dendritic cells, to uptake bacterial by-products diffusing through dentin tubules and evolve pathogen-specific innate and adaptive responses (Durand et al., 2006). However, whether odontoblasts are able to directly destroy intradentinal or peripheral pulp-reaching microorganisms by producing antibacterial agents, without the intervention of immune cells, it is largely unknown. Nitric oxide is a highly diffusible, antimicrobial free radical generated and released by many cell types in inflammatory conditions to kill or inhibit the replication of a variety of microorganisms. NO antimicrobial activity is primarily due to NO reactivity with superoxide anion to form highly cytotoxic peroxynitrite, S-nitrosylation of thiol residues that changes protein conformation, inactivation of enzymes by disruption of iron centers, DNA damage, and membrane lipid peroxidation (Guzik et al., 2003). In this report we provide evidence that odontoblasts differentiated *in vitro* from human dental pulp explants are able to synthesize large amounts of NOS2 and produce NO with antibacterial activity upon TLR2 activation. We first observed that *NOS1*, *NOS2*, and *NOS3* gene expression was significantly increased in Pam2CSK4-stimulated cells compared to controls, *NOS2* being the most up-regulated gene. In agreement with this finding, NOS2 protein was clearly up-regulated in Pam2CSK4-stimulated cells, suggesting its involvement in NO production by these cells. Conversely, NOS1 and NOS3 isoforms, although clearly found in brain and endothelial cell extracts, respectively, were not detected in our Western blot analysis in stimulated or control samples. Accordingly, we speculate that most, if not all, of NO production by Pam2CSK4-stimulated odontoblast-like cells is due to the induction of NOS2, as shown in macrophages stimulated *in vitro* with lipopolysaccharide, another pattern recognition receptor ligand (Denlinger et al., 1996; MacMicking et al., 1997; Guzik et al., 2003).

We found that NO was produced to levels (several tenths of micromoles/liter) similar to those observed in macrophages *in vitro* stimulated with LPS or *Staphylococcus aureus* LTA, or with an association of *Streptococcus mutans* LTA and IFN-γ (Denlinger et al., 1996; Matsuno et al., 1998; Hong et al., 2014). This indicates that the responsivity of odontoblasts upon TLR2 activation, in terms of NO production, is comparable to that of specialized immune cells. To our knowledge, NO concentrations have not been reported in inflamed pulps. However, those we measured in Pam2CSK4-stimulated odontoblast-like cell supernatants were in the range of the concentrations found in periapical exudates from infected human root canals (Shimauchi et al., 2001). This indicated that NOS2-dependent NO amounts produced by TLR2-activated odontoblast-like cells is biologically relevant. We observed here that the number of *Streptococcus mutans* colony-forming units was significantly reduced by NO in stimulated odontoblast-like cells compared to control ones, suggesting the role of NO produced by odontoblasts in the fight against cariogenic bacteria.

We then detected immunohistochemically the NOS2 protein in odontoblasts of inflamed pulps situated beneath dentin caries lesions, whereas it was not present in odontoblasts from healthy ones. This result is in accordance with the fact that NOS2 is not expressed in resting cells, but is only synthesized upon cell activation (Coleman, 2001). Odontoblasts from healthy teeth were also found to be negative for NOS2 (Kawanishi et al., 2004; Kawashima et al., 2005; Mei et al., 2007). NOS2 synthesis was increased in inflamed pulps beneath caries lesions or when inflammation was experimentally induced with bacterial by-products (Kawanishi et al., 2004; Kawashima et al., 2005; Mei et al., 2007; Korkmaz et al., 2011), underscoring the importance of this enzyme in NO production in pulp inflammatory conditions. We observed NOS2 immunoreactivity in odontoblast and subodontoblast cells of the peripheral inflamed pulp, as previously reported in human teeth (Korkmaz et al., 2011). This indicates that odontoblasts are not the only cells involved in the fight again dentin-invading microorganisms. Other cells might also include leukocytes, as previously shown (Guzik et al., 2003). We do not exclude that NOS1 and NOS3 could contribute to the high NO production in the odontoblast layer of bacteria-challenged inflamed pulps. However, several data suggests that such a contribution, if any, would be limited since NOS1 and NOS3 are only able to produce very low concentrations of NO (nanomolar range) compared to NOS2 (micromolar) (Coleman, 2001). The results presented here support the view that NOS2 is a key player in the production of physiologically relevant NO amounts by odontoblasts in bacteria-challenged inflamed pulps. NO could have deleterious effects on odontoblasts themselves. However, these effects might be limited since soluble guanylate cyclase, the receptor that mediates NO effects inside the cell, is decreased in odontoblasts in inflamed human pulps compared to healthy ones (Korkmaz et al., 2011). This indicates that the NO present in the odontoblast layer would rather act on neighboring cells and/or, as suggested by the present study, on dentin-invading bacteria. In this context, NO production by odontoblasts might modulate neurotransmission from deep dentin up to nerve endings present in the proximal dentin or the pulp periphery and/or might contribute to the regulation of the vascular tone of adjacent vessels. The large amount of NO synthesized by odontoblast NOS2 under pathological conditions might thus have an analgesic effect but might also dilate local blood vessels (McCormack and Davies, 1996; Di Nardo Di Maio et al., 2004).

NO has been recently proposed for a clinical use in the treatment of periodontal diseases, because of its drastic reduction of the viability of periodontopathogens (Backlund et al., 2014). Our results also demonstrated an antibacterial effect for NOS2-dependent production of NO by odontoblast-like cells. In the caries context, evidence of NO effects in animal models with cariogenic bacteria is warranted before envisaging the clinical use of NO to prevent dentin and pulp tissue colonization and subsequent irreversible pulpitis and necrosis.

Finally, studies have shown that NO can induce the differentiation of several cell types including osteoblastic, neuronal and endothelial cells (Beltran-Povea et al., 2015). It may also play a part in odontoblast differentiation and the subsequent formation of reparative dentin, notably by augmenting osteocalcin synthesis and alkaline phosphatase activity (Mei et al., 2007; Yasuhara et al., 2007). So NO donors could be use in regenerative dentistry as adjuvants to promote the mineralization events that lead to dentin or bone tissue formation. Additional studies using our culture model and others are required to test this hypothesis.

In summary, we report for the first time that NO with antibacterial activity is produced by TLR2-activated human odontoblast-like cells. Further studies are needed to determine the potential beneficial effect of this molecule on the reduction of human dental pulp inflammation and the promotion of tissue healing and regeneration.

### Conflict of interest statement

The authors declare that the research was conducted in the absence of any commercial or financial relationships that could be construed as a potential conflict of interest.

## References

[B1] Arthur J. S. Ley S. C. (2013). Mitogen-activated protein kinases in innate immunity. Nat. Rev. Immunol. 13, 679–692. 10.1038/nri3495 23954936

[B2] Backlund C. J. Sergesketter A. R. Offenbacher S. Schoenfisch M. H. (2014). Antibacterial efficacy of exogenous nitric oxide on periodontal pathogens. J. Dent. Res. 93, 1089–1094. 10.1177/0022034514529974 25139363 PMC4293763

[B3] Beltran-Povea A. Caballano-Infantes E. Salguero-Aranda C. Martín F. Soria B. Bedoya F. J.. (2015). Role of nitric oxide in the maintenance of pluripotency and regulation of the hypoxia response in stem cells. World J. Stem Cells. 7, 605–617. 10.4252/wjsc.v7.i3.605 25914767 PMC4404395

[B4] Bjorndal L. Mjör I. A. (2001). Pulp–dentin biology in restorative dentistry. Part 4: dental caries—characteristics of lesions and pulpal reactions. Quintessence Int. 32, 717–736. 11695140

[B5] Bleicher F. Richard B. Thivichon-Prince B. Farges J.-C. Carrouel F. (2015). Odontoblasts and dentin formation, in Stem Cell Biology and Tissue Engineering in Dental Sciences, eds Vishwakarma A. Sharpe P. Songtao S. Ramalingam M. (London: Elsevier), 379–395.

[B6] Bogdan C. (2001). Nitric oxide and the immune response. Nat. Immunol. 2, 907–916. 10.1038/ni1001-907 11577346

[B7] Carrouel F. Staquet M. J. Keller J. F. Baudouin C. Msika P. Bleicher F.. (2013). Lipopolysaccharide-binding protein inhibits Toll-like receptor 2 activation by lipoteichoic acid in human odontoblast-like cells. J. Endod. 39, 1008–1014. 10.1016/j.joen.2013.04.020 23880268

[B8] Coleman J. W. (2001). Nitric oxide in inmmunity and inflammation. Int. Immunopharmacol. 1, 1397–1406. 10.1016/s1567-5769(01)00086-8 11515807

[B9] Cooper P. R. Holder M. J. Smith A. J. (2014). Inflammation and regeneration in the dentin-pulp complex: a double-edged sword. J. Endod. 40, S46–S51. 10.1016/j.joen.2014.01.021 24698693

[B10] Couble M. L. Farges J. C. Bleicher F. Perrat-Mabillon B. Boudeulle M. Magloire H. (2000). Odontoblast differentiation of human dental pulp cells in explant cultures. Calcif. Tissue Int. 66, 129–138. 10.1007/pl00005833 10652961

[B11] Denlinger L. C. Fisette P. L. Garis K. A. Kwon G. Vazquez-Torres A. Simon A. D.. (1996). Regulation of inducible nitric oxide synthase expression by macrophage purinoreceptors and calcium. J. Biol. Chem. 21, 337–442. 8550583 10.1074/jbc.271.1.337

[B12] Di Nardo Di Maio F. Lohinai Z. D'Arcangelo C. De Fazio P. E. Speranza L. De Lutiis M. A.. (2004). Nitric oxide synthase in healthy and inflamed human dental pulp. J. Dent. Res. 83, 312–316. 10.1177/154405910408300408 15044505

[B13] Durand S. H. Flacher V. Roméas A. Carrouel F. Colomb E. Vincent C.. (2006). Lipoteichoic acid increases TLR and functional chemokine expression while reducing dentin formation in in vitro differentiated human odontoblasts. J. Immunol. 176, 2880–2887. 10.4049/jimmunol.176.5.2880 16493045

[B14] Farges J. C. Alliot-Licht B. Baudouin C. Msika P. Bleicher F. Carrouel F. (2013). Odontoblast control of dental pulp inflammation triggered by cariogenic bacteria. Front. Physiol. 4:326. 10.3389/fphys.2013.00326 24273514 PMC3823031

[B15] Farges J. C. Carrouel F. Keller J. F. Baudouin C. Msika P. Bleicher F.. (2011). Cytokine production by human odontoblast-like cells upon Toll-like receptor-2 engagement. Immunobiology 216, 513–517. 10.1016/j.imbio.2010.08.006 20850890

[B16] Farges J. C. Keller J. F. Carrouel F. Durand S. H. Romeas A. Bleicher F.. (2009). Odontoblasts in the dental pulp immune response. J. Exp. Zool. Mol. Dev. Evol. 312B, 425–436. 10.1002/jez.b.21259 19067439

[B17] Guzik T. J. Korbut R. Adamek-Guzik T. (2003). Nitric oxide and superoxide in inflammation and immune regulation. J. Physiol. Pharmacol. 54, 469–487. 10.1177/154405910408300408 14726604

[B18] Hahn C. L. Liewehr F. R. (2007). Innate immune responses of the dental pulp to caries. J. Endod. 33, 643–651. 10.1016/j.joen.2007.01.001 17509400

[B19] Hong S. W. Baik J. E. Kang S. S. Yun C. H. Seo D. G. Han S. H. (2014). Lipoteichoic acid of Streptococcus mutans interacts with Toll-like receptor 2 through the lipid moiety for induction of inflammatory mediators in murine macrophages. Mol. Immunol. 57, 284–291. 10.1016/j.molimm.2013.10.004 24216318

[B20] Kawanishi H. N. Kawashima N. Suzuki N. Suda H. Takagi M. (2004). Effects of an inducible nitric oxide synthase inhibitor on experimentally induced rat pulpitis. Eur. J. Oral Sci. 112, 332–337. 10.1111/j.1600-0722.2004.00139.x 15279652

[B21] Kawashima N. Nakano-Kawanishi H. Suzuki N. Takagi M. Suda H. (2005). Effect of NOS inhibitor on cytokine and COX2 expression in rat pulpitis. J. Dent. Res. 84, 762–767. 10.1177/00220345990780100301 16040737

[B22] Keller J. F. Carrouel F. Colomb E. Durand S. H. Baudouin C. Msika P.. (2010). Toll-like receptor 2 activation by lipoteichoic acid induces differential production of pro-inflammatory cytokines in human odontoblasts, dental pulp fibroblasts and immature dendritic cells. Immunobiology 215, 53–59. 10.1016/j.imbio.2009.01.009 19250704

[B23] Keller J. F. Carrouel F. Staquet M. J. Kufer T. A. Baudouin C. Msika P.. (2011). Expression of NOD2 is increased in inflamed human dental pulps and lipoteichoic acid-stimulated odontoblast-like cells. Innate Immun. 17, 29–34. 10.1177/1753425909348527 19880660

[B24] Korkmaz Y. Lang H. Beikler T. Cho B. Behrends S. Bloch W.. (2011). Irreversible inflammation is associated with decreased levels of the alpha1-, beta1-, and alpha2-subunits of sGC in human odontoblasts. J. Dent. Res. 90, 517–522. 10.1177/0022034510390808 21212316

[B25] Law A. S. Baumgardner K. R. Meller S. T. Gebhart G. F. (1999). Localization and changes in NADPH-diaphorase reactivity and nitric oxide synthase immunoreactivity in rat pulp following tooth preparation. J. Dent. Res. 78, 1585–1595. 10.1177/00220345990780100301 10520963

[B26] Love R. M. Jenkinson H. F. (2002). Invasion of dentinal tubules by oral bacteria. Crit. Rev. Oral Biol. Med. 13, 171–183. 10.4049/jimmunol.176.5.2880 12097359

[B27] MacMicking J. Xie Q. W. Nathan C. (1997). Nitric oxide and macrophage function. Annu. Rev. Immunol. 15, 323–350. 10.1146/annurev.immunol.15.1.323 9143691

[B28] Matsuno R. Aramaki Y. Arima H. Adachi Y. Ohno N. Yadomae T.. (1998). Contribution of CR3 to nitric oxide production from macrophages stimulated with high-dose of LPS. Biochem. Biophys. Res. Commun. 244, 115–119. 10.1006/bbrc.1998.8231 9514898

[B29] McCormack K. Davies R. (1996). The enigma of potassium ion in the management of dentine hypersensitivity: is nitric oxide the elusive second messenger? Pain 68, 5–11. 10.1016/s0304-3959(96)03142-9 9251993

[B30] Mei Y. F. Yamaza T. Atsuta I. Danjo A. Yamashita Y. Kido M. A.. (2007). Sequential expression of endothelial nitric oxide synthase, inducible nitric oxide synthase, and nitrotyrosine in odontoblasts and pulp cells during dentin repair after tooth preparation in rat molars. Cell Tissue Res. 328, 117–127. 10.1007/s00441-005-0003-5 17216200

[B31] Nathan C. (1992). Nitric oxide as a secretory product of mammalian cells. FASEB J. 6, 3051–3064. 10.1016/j.niox.2014.09.040 1381691

[B32] Nussler A. K. Billiar T. R. (1993). Inflammation, immunoregulation, and inducible nitric oxide synthase. J. Leukoc. Biol. 54, 171–178. 7689630

[B33] Shimauchi H. Takayama S. I. Narikawa-Kiji M. Shimabukuro Y. Okada H. (2001). Production of interleukin-8 and nitric oxide in human periapical lesions. J. Endod. 27, 749–752. 10.1097/00004770-200112000-00009 11771582

[B34] Silva-Mendez L. S. Allaker R. P. Hardie J. M. Benjamin N. (1999). Antimicrobial effect of acidified nitrite on cariogenic bacteria. Oral Microbiol. Immunol. 14, 391–392. 10895698 10.1034/j.1399-302x.1999.140612.x

[B35] Staquet M. J. Carrouel F. Keller J. F. Baudouin C. Msika P. Bleicher F.. (2011). Pattern-recognition receptors in pulp defense. Adv. Dent. Res. 23, 293–301. 10.1177/0022034511405390 21677082

[B36] Staquet M. J. Durand S. H. Colomb E. Roméas A. Vincent C. Bleicher F.. (2008). Different roles of odontoblasts and fibroblasts in immunity. J. Dent. Res. 87, 256–261. 10.1177/154405910808700304 18296610

[B37] Veerayutthwilai O. Byers M. R. Pham T. T. Darveau R. P. Dalen B. A. (2007). Differential regulation of immune responses by odontoblasts. Oral Microbiol. Immunol. 22, 5–13. 10.1111/j.1399-302x.2007.00310.x 17241164

[B38] Yasuhara R. Suzawa T. Miyamoto Y. Wang X. Takami M. Yamada A.. (2007). Nitric oxide in pulp cell growth, differentiation, and mineralization. J. Dent. Res. 86, 163–168. 10.1177/154405910708600211 17251517

